# Slow and steady—small, but insufficient, changes in food and drink availability after four years of implementing a healthy food policy in New Zealand hospitals

**DOI:** 10.1186/s12916-024-03663-x

**Published:** 2024-10-08

**Authors:** Sally Mackay, Magda Rosin, Bruce Kidd, Sarah Gerritsen, Stephanie Shen, Yannan Jiang, Lisa Te Morenga, Cliona Ni Mhurchu

**Affiliations:** 1 Department of Epidemiology and Biostatistics, School of Population Health, Faculty of Medical and Health Sciences, University of Auckland, Auckland, Private Bag 92019, Auckland Mail Centre, Auckland, 1142 New Zealand; 2 Present Address: School of Population Health, Centre for Translational Health Research: Informing Policy and Practice (TRANSFORM), Faculty of Medical and Health Sciences, University of Auckland, Auckland, Private Bag 92019, Auckland Mail Centre, Auckland, 1142 New Zealand; 3 National Institute for Health Innovation, School of Population Health, Faculty of Medical and Health Sciences, University of Auckland, Auckland, Private Bag 92019, Auckland Mail Centre, Auckland, 1142 New Zealand; 4 Department of Social and Community Health, School of Population Health, Faculty of Medical and Health Sciences, University of Auckland, Auckland, Private Bag 92019, Auckland Mail Centre, Auckland, 1142 New Zealand; 5 Department of Statistics, Faculty of Science, University of Auckland, Auckland, New Zealand; 6 Research Centre for Hauora and Health, Massey University, Wellington, PO Box 756, Wellington, 6140 New Zealand

**Keywords:** Healthy food policy, Voluntary, Hospital, Audit, Employees, Food environment, Nutrition policy

## Abstract

**Background:**

A voluntary National Healthy Food and Drink Policy (the Policy) was introduced in public hospitals in New Zealand in 2016. This study assessed the changes in implementation of the Policy and its impact on providing healthier food and drinks for staff and visitors in four district health boards between 1 and 5 years after the initial Policy introduction.

**Methods:**

Repeat, cross-sectional audits were undertaken at the same eight sites in four district health boards between April and August 2017 and again between January and September 2021. In 2017, there were 74 retail settings audited (and 99 in 2021), comprising 27 (34 in 2021) serviced food outlets and 47 (65 in 2021) vending machines. The Policy’s traffic light criteria were used to classify 2652 items in 2017 and 3928 items in 2021. The primary outcome was alignment with the Policy guidance on the proportions of red, amber and green foods and drinks (≥ 55% green ‘healthy’ items and 0% red ‘unhealthy’ items).

**Results:**

The distribution of the classification of items as red, amber and green changed from 2017 to 2021 (*p* < 0.001) overall and in serviced food outlets (*p* < 0.001) and vending machines (*p* < 0.001). In 2021, green items were a higher proportion of available items (20.7%, *n* = 815) compared to 2017 (14.0%, *n* = 371), as were amber items (49.8%, *n* = 1957) compared to 2017 (29.2%, *n* = 775). Fewer items were classified as red in 2021 (29.4%, *n* = 1156) than in 2017 (56.8%, *n* = 1506). Mixed dishes were the most prevalent green items in both years, representing 11.4% (*n* = 446) of all items in 2021 and 5.5% (*n* = 145) in 2017. Fewer red packaged snacks (11.6%, *n* = 457 vs 22.5%, *n* = 598) and red cold drinks (5.2%, *n* = 205 vs 12.5%, *n* = 331) were available in 2021 compared to 2017. However, at either time, no organisation or setting met the criteria for alignment with the Policy (≥ 55% green items, 0% red items).

**Conclusions:**

Introduction of the Policy improved the relative healthiness of food and drinks available, but the proportion of red items remained high. More dedicated support is required to fully implement the Policy.

**Supplementary Information:**

The online version contains supplementary material available at 10.1186/s12916-024-03663-x.

## Background

In New Zealand (NZ), diets high in energy, sodium, saturated fat and sugar and low in fruit and vegetables and high body mass index are the leading causes of total health loss [1]. In 2023, less than half of NZ adults consumed the recommended amount of fruit and only 11% consumed the recommended servings of vegetables daily [2], and in 2019, 29% of the household food budget was spent on food prepared outside of home [3] which in NZ is predominantly unhealthy [4]. Overall, unhealthy food environments are well recognised as a major cause of poor diets and health [5] and dietary interventions targeted at personal levels without concurrent initiatives in the food environments are likely to produce only small and temporary positive changes in health outcomes [6]. As adults spend considerable time in a workplace, providing healthier food and drink options is important to promote health and wellbeing [7], particularly in health organisations, which should role-model healthy eating. The voluntary National Healthy Food and Drink Policy (the Policy) is one of a small number of government-endorsed actions to improve NZ food environments.

The Policy was introduced in NZ in 2016 to support and encourage the provision of healthier food and drink options for staff and visitors in hospitals and public health sector organisations [8]. The Policy was developed by the National District Health Board Food and Drink Environments Network (the Network), an alliance of nutrition, dietetic, food service and public health representatives from district health boards (DHBs) across NZ along with the Ministry of Health and a university academic (author Ni Mhurchu). At the time this study was conducted, there were 20 DHBs in NZ, which were responsible for providing or funding the provision of health services in their districts. A major restructure of the health service occurred in 2022 and the DHBs were disestablished and organised into four regions under a newly established national Health NZ agency [9].

The first edition of the Policy was published in September 2016 with the intention that DHBs would implement the Policy over a 2-year period [8]. The Policy applies to all food and drinks sold or provided on the premises or on behalf of the organisation. Patient meals, meals on wheels and items brought in for own consumption by staff and visitors are not covered by the Policy. The second edition of the Policy was published in September 2019, with minor changes made to the criteria used to categorise food and drink items to make the Policy more practical to implement, although the overarching principles remained the same.

The Policy provides guidance based on the Eating and Activity Guidelines for NZ Adults [10]. A colour-coded system is used to classify foods as red, amber or green. Green foods and drinks are ‘consistent with the healthy food and drink principles’, ‘reflect a variety of foods from the four core food groups’ (fruit and vegetables, grain foods, milk and milk products and protein foods) and ‘are low in saturated fat, added sugar and added salt, and are mostly whole and less processed’. Amber foods and drinks are ‘not considered part of an everyday diet but may have some nutritive value’. Red foods and drinks are ‘of poor nutritional value and are high in saturated fat, added sugar, and/or added salt’ and include items such as sugar-sweetened beverages, confectionery and deep-fried foods. The Policy states that of all choices available for consumption, green items should predominate (at least 55%), the remaining choices should be amber items (less than 45%), while red items are not permitted (0%). Most packaged foods need to meet set nutrient criteria standards (e.g. a front-of-pack Health Star Rating (HSR) of at least 3.5 stars of 5 possible stars). The HSR is a voluntary front-of-pack labelling system that rates the overall nutritional profile of packaged foods [11]. When the Policy was launched, no tools or resources were available to support the Policy [12], though some support was available to food providers through the Network [13].

During 2021 and 2022, a comprehensive, independently funded evaluation of the implementation of the Policy (the HealthY Policy Evaluation (HYPE)) was conducted in 19 of the 20 DHBs [14]. The 2021/22 audit found that no organisation met the criteria for alignment with the Policy and that 38.9% of food/drink items were rated red (not permitted), 22.1% were green, with the remaining 38.9% classified as amber. Additionally, data existed from a 2017 independent study that assessed the implementation of the Policy in the four Northern DHBs (Northland, Waitematā, Auckland and Counties Manukau), but not in the remaining 16 DHBs. Three of the DHBs (Waitematā, Auckland and Counties Manukau) adopted the National Healthy Food and Drink Policy in 2016. Northland DHB had their own policy which reflected the national Policy except that sugar-free versions of soft drinks were not permitted.

The four DHBs reported on in this paper provide healthcare services to 43% of the total NZ population which in 2022 was estimated to be 2,221,700 people [15]. In 2023, the four Northern DHBs employed approximately 351,200 staff (36.5% of the total DHB workforce) and therefore new workplace policies have the potential to reach a large workforce [16] and positively impact on dietary habits.

The aim of this study was to assess changes in the implementation of the voluntary National Healthy Food and Drink Policy in four DHBs between 2017 and 2021 and evaluate the impact on the healthiness of food and drinks (defined by red, amber and green categories in the Policy) provided to staff and visitors.

## Methods

### Design and settings

This was a repeat, cross-sectional observational study in food retail settings in four DHB regions of the Northern region of NZ. Each DHB had multiple sites (hospitals or clinic sites) with multiple food outlets operating independently in each site. All hospital and clinic sites covered by the four DHBs were included, with one exception. One site, a small rural hospital, was not included, as it was not audited in 2017 due to the time required to travel to the remote location. The audit included all public-access retail outlets at each site that provided foods or drinks for staff or visitors. Food outlets not audited, and thus excluded from the analysis in both 2017 and 2021, were private areas of the hospital/clinic that researchers were not permitted to access. Food outlets were categorised as ‘serviced food outlets’ where the customer is served by a person (cafés, cafeterias, kiosks, coffee carts, sushi outlets, gift shops, fundraisers and pop-up markets) and as ‘vending machines’ where the food and drink service is automated. The following exclusions applied to the audited foods and drinks: inpatient meals, foods and drinks brought in by staff or visitors for their own consumption and hot drinks, as these were not within the Policy’s scope.

### Data collection

The data collection methods for the 2021 audit have been described in detail elsewhere [14] and summarised in this study. Cross-sectional audits of food and drink availability were conducted in 2017 (April to August) using a smartphone where two research assistants took photographs of all individual food and beverage products. In 2021 (January to September), two research assistants and two student dietitians used a customised electronic data collection tool to record all individual foods and drinks available using a tablet or smartphone [17].

The time of day that the audits took place was generally consistent across institutions and food outlets to capture the most extensive selection of foods and drinks on offer at a single time (usually between mid-morning and the start of lunch service). Each food retail outlet was considered a separate setting and all unique products available in singular outlets were collected, regardless of whether they were also available in another food retail outlet.

The following data was recorded either from the photographs (2017) or directly in the digital audit tool (2021): product name or description, brand or manufacturer name, serving size and/or product size (weight in grams or volume in millilitres, either as stated on the product or by weighing with a kitchen scale), nutrition information from the Nutrition Information Panel (NIP) and ingredient list. The nutrition information collected was sodium, saturated fat, total sugars, energy, fibre and protein content per 100 g or 100 mL and per serve where available. If nutrition information was not available or the NIP was not visible on photographs, then it was sourced from manufacturer or supermarket websites, Nutritrack (database of NZ packaged food products) [18] or suppliers. In both audits, for foods or drinks prepared onsite, in the first instance, recipes were sought from the food providers, and, if unavailable, a similar generic food, drink or recipe was used. For products where nutrition data was not available, the research team matched food or drink items to an appropriate substitute in the NZ food composition tables [19]. Serving size was collected as it was required for classification of items in some food and drink policy categories as red, amber and green, in particular bakery items and cold drinks.

The Policy includes eight food categories and one drink category, which altogether encompass 43 food and drink subcategories. One main food category was separated into two main categories for analysis: ‘legumes, nuts, seeds’ (most products in this category are snack packs of nuts and seeds) and ‘fish and other seafood, eggs, poultry and red meat’ (most products in this category consumed as part of a meal). The resulting ten main food/drink categories used in this analysis were bakery items; cold drinks; fats and oils, spreads, sauces, dressings and condiments; fish and other seafood, eggs, poultry and red meat; grain foods; legumes, nuts, seeds; milk and milk products; mixed dishes; packaged snack foods; and vegetables and fruit. Each product was assigned to one main food/drink category and one subcategory (described in Additional file 1: Table S1).

### Analysis

Data from eight sites in the four DHBs were included in the analysis in both years. In total, the 8 hospital/clinic sites audited comprised 74 individual retail settings in 2017 and 99 in 2021 (Table 1). Data from the rural hospital collected in 2021, but not in the 2017 study, were excluded to maintain consistency between years. Additionally in 2021, one fast-food chain outlet did not consent to the data collection, so this outlet was also removed from the 2017 dataset (57 foods/drinks removed). In 2017, there was a small grocery store at one DHB (but no longer operating in 2021) which sold ready-to-eat products, such as single-serve packaged items (e.g. muesli bar, single-serve drink), which were included in the analysis. However, products sold by this store that required further preparation or cooking outside of the hospital setting (e.g. dried legumes, baking ingredients and raw grains) were excluded from the analysis (*n* = 103, 2.5% of all audit products) as they could not be prepared and consumed on the premises by staff or visitors, and thus were out of scope of the Policy. Note, hot drinks are out of scope of the Policy. Additionally, products were excluded if insufficient information was available to classify them.

**Table 1:** Characteristics of participating organisations in 2017/2021

DHB	Population covered (2022)	Number of operating sites	Number of food retail settings		Number of serviced food outlets^a^		Number of vending machines	
			2017	2021	2017	2021	2017	2021
Northland	201,500	1	4	5	3	3	1	2
Waitemata	633,500	2	22	18	6	7	16	11
Auckland	781,600	3	25	48	8	12	17	36
Counties Manukau	605,100	2	23	28	10	12	13	16
Total	2,221,700	8	74	99	27	34	47	65

^a^Cafés, cafeterias, kiosks, coffee carts, sushi outlets, gift shops, fundraisers, pop-up markets

Within-retail outlet changes were assessed for serviced food outlets that were in the same location during the 2017 and 2021 audits. Outlets with 20 or fewer items in one audit were excluded. This analysis was not conducted for vending machines due to the small number of products available in each machine, changes in their location and the higher number of vending machines audited in 2021 compared to 2017.

The second edition of the Policy that was released in 2019 was used to categorise foods and drinks into food/drink categories and subcategories and classify them as red, amber and green for the 2017 and 2021 datasets. The updated Policy had overall minor changes in categorisation criteria for food and drink items. However, there were notable changes in mixed dishes category. In the first edition, the requirements for meals in the green category were to be prepared only with green-category ingredients and, for some subcategories, to also have ≥ 50% of the meal as vegetable or fruit. These criteria were changed in the second edition to make the Policy more practical to implement allowing mixed dishes to contain ≤ 25% of amber ingredients and needing to contain some vegetables or fruit. Therefore, only the second edition criteria for the Policy were used in this study.

The product description, ingredient list, nutrition information and serving size information was used to categorise items according to one of the nine food categories or a drink category as outlined in the Policy and to classify each item as red, amber or green. Nutrition data were also used to estimate the HSR of a product using the publicly available HSR calculator [11] where HSR was required to classify it with the traffic light system. In 2017, food and drink product information were recorded directly from photographs into an excel spreadsheet and categorised manually. Products in the 2017 dataset were then reclassified using the second edition of the Policy. Authors SM and MR identified the differences in the classification and the methods for reclassification. One research assistant (CG) conducted the reclassification, consulting SM and MR about any products difficult to classify. In 2021, the digital audit tool had algorithms inbuilt for selecting the food/drink category and subcategory, estimating HSR from nutrition information and using this information for classification as red, amber or green. Random quality checks on a sample of 10% of products were carried out in the digital audit tool by two research assistants who reviewed and agreed on changes (SS and BK), and the entire dataset was extracted as an excel file. Further comprehensive quality checks on both datasets were undertaken by SM and MR to check consistency in the food and drink categorisation and traffic light classification within and between datasets; any duplicate products in the same food setting were removed.

Statistical analyses were performed using SAS version 9.4. All unique products within an individual retail setting were included in analysis and presented as frequencies and percentages between categories and years. The differences in healthiness rating (i.e. proportions of red, amber and green items) within each category, within each setting and overall were tested between 2017 and 2021 using the chi-square tests. Statistical significance level was set at *p* < 0.05 (two-sided).

## Results

### Characteristics of participating organisations and food outlets

There were 74 individual retail settings in 2017 and 99 in 2021 (Table 1). Approximately one-third of settings were serviced food outlets (*n* = 27, 36.5% in 2017 and *n* = 34, 34.3% in 2021). Almost two-thirds of the retail settings were vending machines (*n* = 47, 63.5% in 2017 and *n* = 65, 65.7% in 2021). There were 19 serviced outlets in the same location in 2017 and 2021 for the within-retail analysis.

### Products and categories

Information was collected for 2655 foods and drinks in 2017 and 3991 in 2021. Three products (0.1%) in 2017 and 63 products (1.6%) in 2021 that could not be classified as red, amber or green due to incomplete product information were excluded. The final audit sample included 2652 products in 2017 and 3928 in 2021 (Table 2). The food/drink categories with the most items in both years were mixed dishes (27.5% of items in 2017, *n* = 728; 28.0% of items in 2021, *n* = 1100), packaged snack foods (23.5%, *n* = 624; 15.9%, *n* = 625), cold drinks (19.2%, *n* = 510; 22.4%, *n* = 881) and bakery items (13.8%, *n* = 365; 15.5%, *n* = 609) (Table 2).

**Table 2:** Availability of items per food category and setting, 2017/2021, number (%) of total products

Category	Serviced food outlets^a^				Vending machines				Total			
	**2017**		**2021**		**2017**		**2021**		**2017**		**2021**	
	***n***	**%**	***n***	**%**	***n***	**%**	***n***	**%**	***n***	**%**	***n***	**%**
Bakery items	294	15.1	490	18.9	71	10.1	119	8.9	365	13.8	609	15.5
Cold drinks	332	17.0	491	19.0	178	25.3	390	29.1	510	19.2	881	22.4
Fats and oils, spreads, sauces, dressings, condiments	46	2.4	98	3.8	0	0	0	0	46	1.7	98	2.5
Fish and other seafood, eggs, poultry, red meat	16	0.8	62	2.4	0	0	0	0	16	0.6	62	1.6
Grain foods	42	2.2	40	1.5	6	0.9	0	0	48	1.8	40	1.0
Legumes, nuts, seeds	45	2.3	61	2.4	93	13.2	240	17.9	138	5.2	301	7.7
Milk and milk products	86	4.4	52	2.0	3	0.4	7	0.5	89	3.4	59	1.5
Mixed dishes	686	35.2	1025	39.6	42	6.0	75	5.6	728	27.5	1100	28.0
Packaged snack foods	316	16.2	123	4.7	308	43.8	502	37.5	624	23.5	625	15.9
Vegetables and fruit	85	4.4	148	5.7	3	0.4	5	0.4	88	3.3	153	3.9
Total	1948	100	2590	100	704	100	1338	100	2652	100	3928	100

^a^Cafés, cafeterias, kiosks, coffee carts, sushi outlets, gift shops, fundraisers, pop-up markets

### Products available by settings

In 2017, two-thirds of all audited items (73.5%, *n* = 1948) were available from serviced food outlets and the remaining 704 items (26.5%) were in vending machines. In 2021, this shifted to a lower proportion of items (65.9%, *n* = 2590) available from serviced food outlets and a higher proportion (34.1%, *n* = 1338) available in vending machines (Table 2). There were more food settings audited in 2021 (99 in 2021, 74 in 2017) which is reflected by more products in the 2021 sample.

Of the products available in vending machines in 2021 and 2017, respectively, most were packaged snack foods (37.5%, *n* = 502; 43.8%, *n* = 308), cold drinks (29.1%, *n* = 390; 25.3%, *n* = 178) and legumes/nuts/seeds (17.9%, *n* = 240; 13.2%, *n* = 93). In contrast, the most common categories in serviced food outlets were mixed dishes (39.6%, *n* = 1025 in 2021; 35.2%, *n* = 686 in 2017), cold drinks (19.0%, *n* = 491; 17.0%, *n* = 332) and bakery items (18.9%, *n* = 490; 15.1%, *n* = 294). In 2021, packaged snack foods represented 4.7% (*n* = 123) of items in serviced food outlets, which was lower than in 2017 (16.2% of items, *n* = 316) (Table 2).

### Change in proportions of red, amber and green category items between 2017 and 2021

**Fig. 1:**
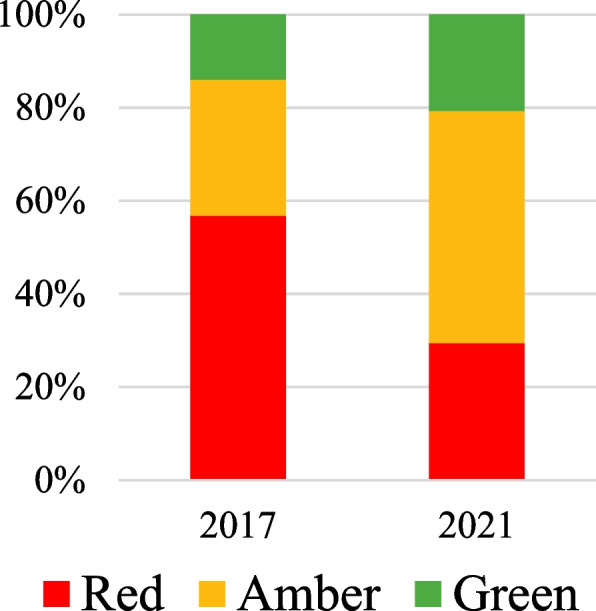
Classification of items as red, amber or green in 2017 and 2021

Overall, there was a significant change in the distribution of food/drink items classified as red (not permitted), amber and green between the two years (*p* < 0.001). The proportion of red items was lower in 2021 (29.4%, *n* = 1156) than in 2017 (56.8%, *n* = 1506) (Table 3, Fig. 1). The proportion of items classified as green was higher in 2021 (20.7%, *n* = 815) than in 2017 (14.0%, *n* = 371), with a similar change observed for amber items (49.8%, *n* = 1957 in 2021 vs 29.2%, *n* = 775 in 2017). None of the 4 DHBs met the Policy criteria of having at least 55% of the foods and drinks available classified as green and no red items available in either 2017 or 2021 (Table 3). In the individual DHBs, the % of items classified as green ranged from 18.6 to 40.1% in 2021 and classified as red from 21.8 to 35.6%. This is a positive shift from 2017 where green items ranged from 8.4 to 22.7% and red from 40.1 to 70.4% of available items.

**Table 3:** Classification of items as red, green or amber by region and organisation in 2017/2021

DHB	Products with classification													
	**2017**							**2021**						
	**Total**	**Red**		**Amber**		**Green**		**Total**	**Red**		**Amber**		**Green**	
	***N***	***n***	**%**	***n***	**%**	***n***	**%**	***N***	***n***	**%**	***n***	**%**	***n***	**%**
Northland	172	69	40.1	64	37.2	39	22.7	202	44	21.8	77	38.1	81	40.1
Waitemata	751	529	70.4	159	21.2	63	8.4	778	188	24.2	408	52.4	182	23.4
Auckland	762	396	52.0	230	30.2	136	17.8	1797	514	28.6	948	52.8	335	18.6
Counties Manukau	967	512	52.9	322	33.3	133	13.8	1151	410	35.6	524	45.5	217	18.9
All DHBs	2652	1506	56.8	775	29.2	371	14.0	3928	1156*	29.4	1957*	49.8	815*	20.7

The Policy states that ≥ 55% of products on offer must fit within green. No red products are permitted

^*^Test of significance *p* < 0.001 between 2017 and 2021. Tests were not conducted on individual DHBs

### Changes by retail setting over time

**Fig. 2:**
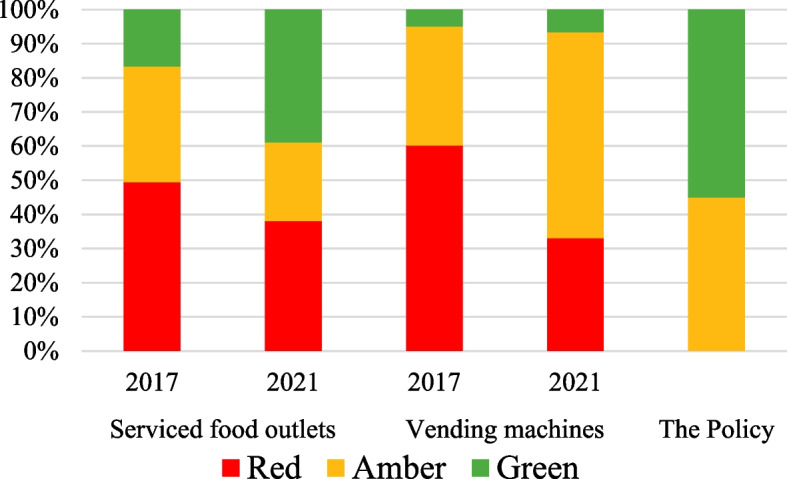
Classification of items as red, amber and green by setting in 2017 and 2021

The distribution of the classification of foods and drinks changed significantly between the two years in both retail outlet categories (Table 4, Fig. 2) (*p* < 0.0001 for both settings). There were fewer red items in 2021 compared to 2017 in serviced food outlets (27.5% of products, *n* = 713 vs 49.5% of products, *n* = 964) and vending machines (33.1%, *n* = 443 vs 77.0%, *n* = 542). There was a corresponding increase in green and amber items in serviced food outlets in 2021 from 2017 (from 16.7%, *n* = 326 to 28.1%, *n* = 727 for green items and from 33.8%, *n* = 658 to 44.4%, *n* = 1150 for amber items). A similar pattern was observed for amber items in vending machines, with an increase from 16.6% of items (*n* = 117) in 2017 to 60.3% (*n* = 807) in 2021. There was little change in green items between the two years (6.6%, *n* = 88 in 2021 and 6.4%, *n* = 45 in 2017) (Table 4).

**Table 4:** Classification of items as red, amber or green by setting in 2017 and 2021

	**Products with classification**														**Level of significance**^**a**^
	**2017**							**2021**							
	**Total**	**Red**		**Amber**		**Green**		**Total**	**Red**		**Amber**		**Green**		
	*N*	*n*	%	*n*	%	*n*	%	*N*	*n*	%	*n*	%	*n*	%	
Serviced food outlets^b^	1948	964	49.5	658	33.8	326	16.7	2590	713	27.5	1150	44.4	727	28.1	< 0.001
Vending machine	704	542	77.0	117	16.6	45	6.4	1338	443	33.1	807	60.3	88	6.6	< 0.001

^a^Test of significance conducted on change in classification (red, amber, green) between the two years (2017, 2021)

^b^Cafés, cafeterias, kiosks, coffee carts, sushi outlets, gift shops, fundraisers, pop-up markets

### Changes within serviced food outlets over time

**Fig. 3:**
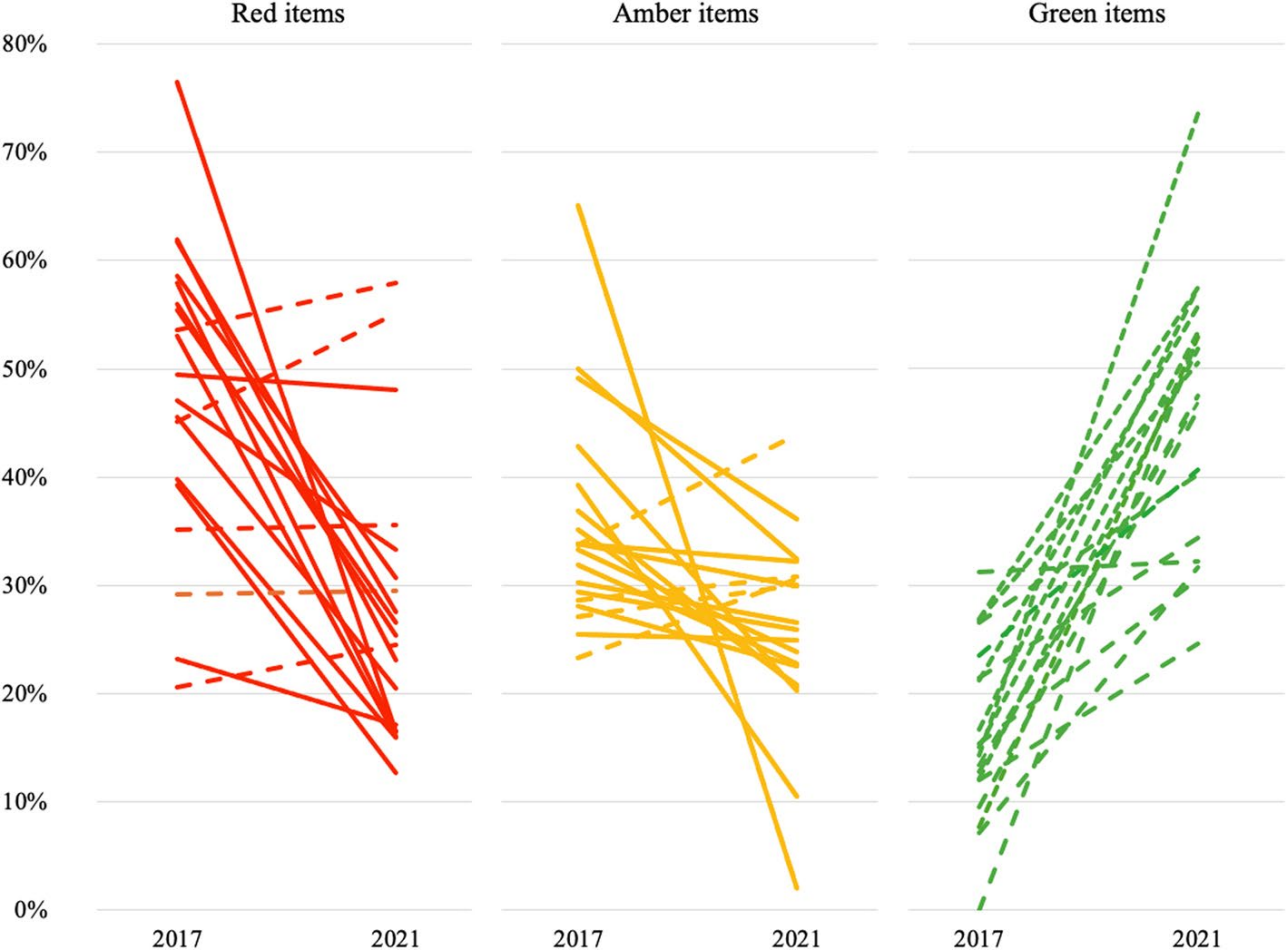
Change in number of items classified as red, amber and green within food service outlets present in 2017 and 2021 audits

Nineteen serviced outlets were in the same location during both audits. In 14 outlets (74%), there was a decrease in the proportion of red items offered by at least 5 percentage points (Fig. 3, Additional file 1: Table S2). In all but one outlet, there was an increase in the proportion of green items offered by at least 5 percentage points. The change in the proportion of amber items offered was mixed with a reduction of amber items offered in 10 outlets (53%) by at least 5 percentage points, an increase in 2 outlets (11%) and minimal change (less than 5 percentage points) in 7 outlets (37%).

### Changes by food/drink category over time

**Fig. 4:**
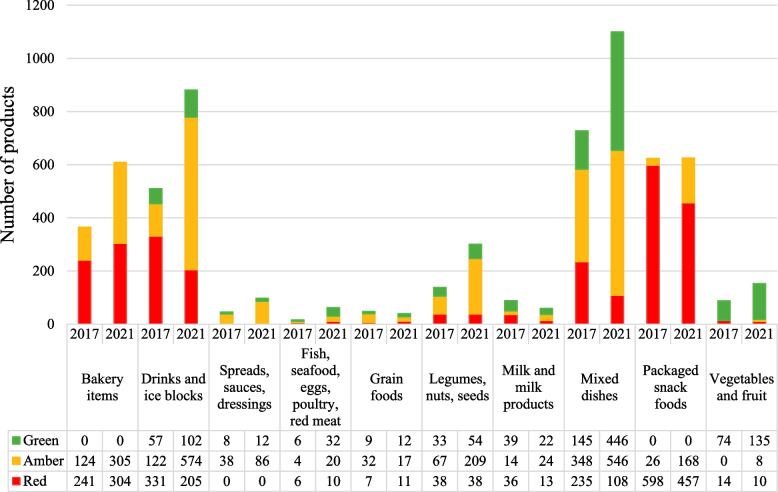
Classification of items as red, amber and green by category in 2017 and 2021

Significant changes in the distribution of red, amber and green items were observed between 2021 and 2017 for seven of the ten food/drink categories with Table 5 showing this as the proportion within the category. Figure 4 and Additional file 1: Table S3 show the proportion as the total of all products. The category with the highest proportion of green products was fruit and vegetables but this made up only 3.4% (*n* = 135) of items in 2021 and 2.8% (*n* = 74) in 2017, with many items in the fruit and vegetable category sold as part of mixed dishes.

**Table 5:** Classification of items as red, amber and green items, by food category in 2017 and 2021

**Category**	**2017**						**2021**						**Level of significance**^a^
	**Red**		**Amber**		**Green**		**Red**		**Amber**		**Green**		
	***n***	**%**	***n***	**%**	***n***	**%**	***n***	**%**	***n***	**%**	***n***	**%**	
Bakery items^b^	241	66	124	34	0	0	304	49.9	305	50.1	0	0	< 0.001
Cold drinks	331	64.8	122	24.3	57	10.9	205	22.6	574	66.1	102	11.4	< 0.001
Fats and oils, spreads, sauces, dressings, condiments	0	0	38	82.6	8	17.4	0	0	86	87.8	12	12.2	0.4051
Fish and other seafood, eggs, poultry, red meat	6	37.5	4	25	6	37.5	10	16.1	20	32.3	32	51.6	0.1679
Grain foods	7	14.6	32	66.7	9	18.8	11	27.5	17	42.5	12	30	0.0733
Legumes, nuts, seeds	38	27.5	67	48.6	33	23.9	38	12.6	209	69.4	54	17.9	< 0.001
Milk and milk products	36	40.4	14	15.7	39	43.8	13	22	24	40.7	22	37.3	0.0018
Mixed dishes	235	32.3	348	47.8	145	19.9	108	9.8	546	49.6	446	40.5	< 0.001
Packaged snack foods^b^	598	95.8	26	4.2	0	0	457	73.1	168	26.9	0	0	< 0.001
Vegetables and fruit	14	15.9	0	0	74	84.1	10	6.5	8	5.2	135	88.2	0.0081

^a^Test of significance conducted on change in classification (red, amber, green) between the two years

^b^There are no criteria for green classification for bakery items or packaged snack foods in the Policy

### Changes in cold drinks and packaged snack foods over time

Further analysis was conducted in two of the categories with the most products (cold drinks and packaged snacks) to understand in which settings changes were occurring (Additional file 1: Table S4). First, the type of cold drinks that were the most available changed. Still or carbonated flavoured drinks made up a larger proportion of the available drinks in 2021 (55.1%, *n* = 485) compared to 38.6% (*n* = 197) in 2017. Correspondingly, juices made up a lower proportion of the drinks available in 2021 (28.4%, *n* = 250) compared to 2017 (36.3%, *n* = 185). Bottled water made up a similar proportion of the drinks category in 2021 (14.1%, *n* = 124) and 2017 (15.1%, *n* = 77), with most waters classified as green (79.8%, *n* = 99 in 2021 and 71.4%, *n* = 55 in 2017).

Second, decreases in the proportion of red cold drinks available were observed for both serviced food outlets (37.3%, *n* = 182, in 2021 from 63.9%, *n* = 212, in 2017) and vending machines (5.1%, *n* = 20, from 66.9%, *n* = 119) (Additional file 1: Table S5). Additionally, a significant increase in the proportion of amber cold drinks was noted for both serviced food outlets (50.4%, *n* = 246, in 2021 from 26.8%, *n* = 89, in 2017) and vending machines (84.1%, *n* = 328 from 18.5%, *n* = 33).

Third, for the packaged snack food category, there was a decrease in the proportion of red items between 2021 and 2017. In 2021, 73.1% (*n* = 457) of all packaged snack foods were classified as red, compared to almost all (95.8%, *n* = 598) packaged snack foods classified as red in 2017 (Table 5). The changes in the classification of packaged snacks available were observed in both serviced food outlets and vending machines (Additional file 1: Table S5). In 2021, 67.5% (*n* = 83) and 74.5% (*n* = 374) of all packaged snack foods were red in serviced food outlets and vending machines, respectively. In comparison, 91.8% (*n* = 290) of packaged snack foods were red in 2017 in serviced food outlets and 100% (*n* = 308) in vending machines. The remaining items were classified as amber (there is no criteria for green classification in the Policy). In 2017, 21% (*n* = 124) of packaged snack foods were classified as confectionary while only one product (0.2%) was in 2021.

## Discussion

We assessed the impact of the voluntary National Healthy Food and Drink Policy (the Policy) on foods and drinks for staff and visitors in hospitals 1 (in 2017) and 5 years (in 2021) after its introduction in four health districts (DHBs) using researcher-led onsite audits. There was an improvement in the healthiness of products available for sale in both serviced food outlets and vending machines from 2017 to 2021. However, none of the four DHBs met the Policy criteria (at least 55% of the foods and drinks available classified as green and no red items available) in either 2017 or 2021. Improvements were seen in all but one of the serviced outlets audited in both years, with a higher proportion of green items and a corresponding lower proportion of red items. Despite the higher proportion and the total number of individual food and drinks available for sale classified as green (healthy) in 2021, and corresponding lower proportion and number of red (unhealthy) items, further implementation and support for food providers are required to meet the Policy criteria.

Even though no organisation fully met the Policy criteria, it is encouraging to observe improvements to the healthiness of food and drink available in the NZ health sector between 2017 and 2021. There may be several reasons for individual DHBs not meeting the Policy criteria, including the Policy being voluntary with no enforcement mechanisms or consequences for non-compliance, which are barriers to implementation noted in the literature [20]. The implementation of the Policy has been supported by the Network members at their respective DHBs, but our engagement with key NZ stakeholders and results from the food provider and Network member interviews conducted as part of the HYPE study indicate that the nutritional support has not been a major part of the Network members’ roles [13]. Other research by our team [12] also found insufficient additional tools or resources allocated to support the implementation and evaluation of the Policy or to engage with food providers and staff and visitors, especially when compared to similar policies in Australia. Interviews with food providers from healthcare facilities in New Zealand in 2022 noted concerns about a decrease in profits following the implementation of the Policy [13]. Food providers perceived that complying with the Policy would result in a business disadvantage (and no apparent business benefits) due to higher costs of some healthier items, possible food wastage from unsold healthier items and because, at most sites, customers could easily purchase less healthy items from food outlets that did not have to comply with the Policy just outside the hospital. The drop in profits after the initial adoption of the Policy that did not recover over time (as reported during interviews by food providers) was not assessed during the HYPE study due to the lack of access to sales data from food providers. Three-quarters of staff and visitors surveyed about food outlets in healthcare organisations in New Zealand reported that foods and drinks were more expensive since introduction of the Policy [21].

A reduction in revenue has been reported in other studies in healthcare settings. Introduction of a healthy food and beverage policy for vending machines in a major health service in Australia found sales of less healthy items significantly decreased but were not fully compensated by sales of healthier items [22], though negotiation of higher commission rates meant there was not change in revenue from vending. After implementation of a mandatory Healthcare Retail Standard in the Scottish National Health Service, overall sales did fall, impacting profits; however, over time, sales increased but did not return to the pre-standard period [23].

Despite a lack of supportive tools and resources, improvements in the healthiness of food and drinks available indicate the effort of food providers and Network members to identify and stock new, healthier products, including those in smaller package sizes particularly for drinks and packaged snack foods, and bakery items, where classification directly or indirectly involves serving/package size. However, the persisting high proportion of red items in these (and most other) categories could reflect the overall poor nutritional quality of NZ packaged food supply [24], difficulty in finding healthier products on the market [20] and the complexity of the Policy’s nutritional criteria, including deciding on the appropriate food category for some items. Several food categories require the front-of-pack HSR value to classify foods as either red, amber or green. HSR is a voluntary labelling scheme in NZ and Australia, with slow uptake by the food industry (available on less than one-third of products) [25], so food providers and Network members manually calculate HSR of many food products, likely increasing the challenge in classifying foods. It is not surprising that only one of seven similar health facility policies in Australia use the HSR in their nutritional criteria [26]. Less complex policy criteria that use the readily available product and nutrition information could facilitate further implementation of the Policy in NZ, and addition of the HSR could be considered only if the HSR labelling scheme were to become mandatory.

There is an indication that manufacturers have been responsive to the introduction of the Policy in NZ [13]. For example, by increasing the range of their products and producing smaller package sizes to meet volume restrictions set in the Policy for amber items. In particular, there was a large shift over the 4 years in drinks available for sale from red to amber. Availability of flavoured waters classified as amber was greater in 2021 (80% of flavoured waters) compared to 2017 (29%). Additionally, two-thirds of fruit/vegetable juices were classified as amber in 2021, compared to most juices classified as red in 2017.

Based on our findings, similar manufacturing trends could be underway to increase amber bakery items and packaged snack foods in the NZ market. There are no green criteria available in the Policy for these two categories so the current Policy criteria could be reviewed to assess if baked items or snacks could align with the principles for green items being part of a healthy diet [8]. For example, in Australian healthcare facilities, lightly salted and flavoured popcorn and muesli and snack bars up to 50 g are considered everyday snacks in the New South Wales Policy [27] and quiches without pastry made with lean meat and/or vegetable fillings are considered green in the Queensland Policy [28].

In recent years, there has been an increase in healthy food and drink policies in healthcare settings [29] but adoption of such policies does not guarantee their full implementation and adherence, which remains problematic worldwide. Direct comparison between studies is challenging since individual policies have different nutritional criteria and the methods of evaluation used by researchers can vary. However, it seems that mandatory policies are most effective at improving the healthiness of food and drinks in public health sector organisations over time. An independent audit of a mandatory scheme, the ‘Scottish Healthcare Retail Standard’, which required all hospital food retail outlets to change the balance of foods products stocked found that all shops achieved compliance and there was an improvement in offerings after 1 year [30]. The authors noted that the consistent approach across hospital shops changed the context in which food purchase decisions are made and that the use of regulatory schemes can bring about change across different types of retailers [30]. A mandatory policy was introduced in health facilities in New South Wales (Healthier Food and Drink Choices for Staff and Visitors) in 2007. An independent audit conducted in 2013 found the proportion of healthier beverage vending machine slots increased significantly after introduction of a policy but only minor changes occurred in the availability of healthier food or drink choices in food outlets [31]. In Western Australia, the Healthy Options Policy was introduced in 2008 and Western Australia Health services were requested to become fully compliant by October 2018 with support provided from health service providers. An audit conducted soon after this deadline demonstrated improvements and progression towards policy compliance since an earlier audit in 2016, but not full compliance [32].

### Policy implications and recommendations

This research indicates that a voluntary policy can effect some positive change in a public health sector food environment, but a mandatory policy with dedicated support for implementation is needed for retailers to adhere to the Policy. This will require leadership and funding from the relevant government agency to support implementation actions, such as more collaboration and engagement with food providers, suppliers and manufacturers, as well as development and provision of resources, monitoring and enforcement of the Policy. The diversity of barriers, facilitators and means to overcome challenges reported in the literature [20] implies a range of supportive actions and tools are needed for successful policy implementation. Some of these actions are underway in NZ, with the Policy currently under review to make it easier for foodservice providers to interpret and implement and to ensure it meets the needs of the staff and visitors to NZ healthcare facilities. Additionally, a searchable online database of packaged foods and drinks is under development, and this tool could be used by food providers to check products’ traffic light classification and to identify healthier options on the NZ market. Repeat onsite audits of all or a subsample of foodservice outlets will be necessary in the future across NZ to assess the effectiveness and impact of the updated Policy and to evaluate if additional support and tools facilitated its implementation.

### Strengths and limitations

We conducted comprehensive, systematic, onsite audits that included all foods and drinks available in public areas rather than a convenience sample of products or outlets. We applied the same nutrition criteria to assess all products in both datasets and carried out extensive data quality checks, which ensured consistency in the analysis. Additionally, the 2017 and 2021 audits were funded and conducted by independent third parties, so the evaluation design, data collection, analysis and interpretation of our results were impartial.

However, there are some study limitations. First, data from the 2017 evaluation was available for four of the 20 DHBs, allowing us to assess the changes in a limited number of health districts and the results are not representative of NZ. Despite that, our evaluation included three large metropolitan DHBs and one medium size DHB that collectively provide services to 43% of the NZ population (including diverse population groups) and employ 36.5% of the health workforce. Additionally, the three large DHBs were ahead of the other DHBs in developing and implementing a healthy food and drink policy, so our results are valuable for the remaining DHBs and in reviewing the Policy and planning future implementation actions. Second, the 2017 audit was the first instance that food and drink availability data was collected in some DHBs, and no baseline data prior to the Policy introduction exists. The lack of baseline data limits our ability to assess the extent of impact the introduction of the Policy had in the four DHBs and overall in NZ. It is therefore advisable that jurisdictions complete baseline data collection prior to food environment policy introduction. Third, we sometimes relied on product photos to correctly classify foods and drinks, particularly for the 2017 audit. However, to ensure consistency, we systematically categorised all products and conducted extensive quality checks on both datasets. Fourth, the audits captured the availability of products rather than actual sales so we cannot indicate if purchasing of red, amber or green products was different in 2017 and 2021, or whether it has changed since the Policy was first introduced. We assume that higher availability of healthier options can ultimately lead to healthier diets, but we require access to individual items’ sales data to determine if healthier options are being purchased and by whom in order to assess potential health benefits for staff and visitors. We recommend that contracts include requirements for the provision of sales data for such analysis [33]. However availability of healthier options in other workplace cafeterias has been associated with healthier consumption [34, 35] and an earlier study in NZ found that increasing the healthiness of products in hospital vending machines did not affect sales volumes [36]. Fifth, there were more outlets audited in 2021 than in 2017. We believe that this was, at least in part, due to a proliferation of vending machines due to the COVID-19 pandemic to reduce person-to-person contact. Additionally, it is possible that researchers were not allowed to access some vending machines in 2017 but were in 2021. There was also a small increase in the audited serviced food outlets in 2021 due to researchers having access to 2 staff cafes in 2021 but not in 2017; the Policy jurisdiction being clarified to include 3 outlets located on the street by one hospital site in the 2021 audit; and the presence of 2 additional temporary outlets in 2021. We were able to specify the changes in food and drink availability within 19 serviced food outlets, which may be attributed to the Policy, but could also be due to factors such as changes in outlet ownership or operator, variations in food suppliers and the level of support available to food providers to implement the Policy. Lastly, we were unable to assess the effect, if any, the COVID-19 pandemic had on the availability and type of food and drink products in healthcare facilities. The first audit was conducted in 2017 before the COVID-19 pandemic and the second audit in 2021, during the pandemic.

## Conclusion

While none of the four DHBs included in our cross-sectional audits was fully compliant in either 2017 or 2021, there were substantial shifts towards meeting the Policy criteria with less red items and more amber and green options available in 2021 compared to 2017. To increase the pace towards meeting the Policy criteria and observe further positive changes in the health sector food environments, the voluntary National Healthy Food and Drink Policy should be mandatory, endorsed by the NZ government and adequately supported to carry out implementation and evaluation actions.

## Supplementary Information

Additional file 1: Tables S1 to S5 contain a description of the food categories and additional data on the availability and/or classification of items within individual outlets, by category, by subcategories of drinks and within type of setting for cold drinks and packaged foods. Table S1 Policy food and drink categories and subcategories. Table S2 Change in number of items classified as red, amber and green within food service outlets present in 2017 and 2021. Table S3 Classification of items as red, amber and green items, by category in 2017 and 2021, as a percentage of all products. Table S4 Availability of items in the drinks category in 2017 and 2021. Table S5 Classification of cold drinks and packaged foods as red, amber and green items, by setting in 2017 and 2021.

## Data Availability

The participants of this study did not give written consent for their data to be shared publicly due to the commercially sensitive nature of the data. However, the corresponding author may grant access to specific, anonymised datasets upon reasonable request.

## Abbreviations

DHBs: District health boards
HSR: Health Star Rating
HYPE: HealthY Policy Evaluation
NZ: New Zealand
