# Supplementary material for: Slow and steady—small, but insufficient, changes in food and drink availability after four years of implementing a healthy food policy in New Zealand hospitals
Source: BMC Med. 2024 Oct 8;22:437. doi: 10.1186/s12916-024-03663-x (PMC11462770; doi:10.1186/s12916-024-03663-x)
Supplement: Supplementary file 1 — Additional file 1: Tables S1 to S5 contain a description of the food categories and additional data on the availability and/or classification of items within individual outlets, by category, by subcategories of drinks and within type of setting for cold drinks and packaged foods. Table S1 Policy food and drink categories and subcategories. Table S2 Change in number of items classified as red, amber and green within food service outlets present in 2017 and 2021. Table S3 Classification of items as red, amber and green items, by category in 2017 and 2021, as a percentage of all products. Table S4 Availability of items in the drinks category in 2017 and 2021. Table S5 Classification of cold drinks and packaged foods as red, amber and green items, by setting in 2017 and 2021. [file 12916_2024_3663_MOESM1_ESM.docx]

**Table S1: Policy food and drink categories and sub-categories**

| **Category** | **Subcategories** |
| --- | --- |
| Bakery items | Biscuits, muesli bars, pikelets |
|  | Loaf, muffins |
|  | Pies and quiches |
|  | Sausage rolls |
|  | Scones, cakes or dessert |
|  | Slices, friands |
|  | Small pastries |
| Cold drinks | 100% fruit and/or vegetable juices |
|  | Energy drinks  Ice blocks  Milk-based smoothies |
|  | Still or carbonated flavoured drinks and milk drinks (milkshake, liquid breakfast) |
|  | Water |
| Fats and oils, spreads, sauces, dressings, condiments | Butter |
|  | Margarine |
|  | Nut butter |
|  | Sauces (mayonnaise, tomato) and salad dressings |
|  | Savoury condiments |
|  | Sweet condiments (jam, honey, compote) |
| Fish and other seafood, eggs, poultry, red meat | Canned and packaged fish, chicken and meat |
|  | Eggs |
|  | Fresh or frozen fish and seafood, poultry, lean meat |
|  | Processed fish, chicken and meat products |
| Grain foods | Bread |
|  | Breakfast cereals |
|  | Cereal foods |
| Legumes, nuts, seeds | Legumes |
|  | Nuts and seeds |
| Milk and milk products | Cheese |
|  | Cream, sour cream, cream cheese |
|  | Frozen desserts (yoghurt, ice-cream) |
|  | Yoghurt / dairy food |
| Mixed dishes | Packaged mixed dishes including ready-to-eat products |
|  | Sandwiches |
|  | Sushi |
|  | Unpackaged mixed dishes including ready-to-eat products |
| Packaged snack foods | Confectionery |
|  | Other than single ingredient foods listed in other categories |
| Vegetables and fruit | Fruit |
|  | Vegetables |

**Table S2: Change in number of items classified as red, amber and green within food service outlets present in 2017 and 2021 audits**

| **Serviced outlet** | **Products with Classification** | | | | | | | | | | | | | |  |
| --- | --- | --- | --- | --- | --- | --- | --- | --- | --- | --- | --- | --- | --- | --- | --- |
|  | **2017** | | | | | | | **2021** | | | | | | |  |
|  | **Total** | **Red** | | **Amber** | | **Green** | | **Total** | **Red** | | **Amber** | | **Green** | |  |
|  | **N** | **n** | **%** | **n** | **%** | **n** | **%** | **N** | **n** | **%** | **n** | **%** | **n** | **%** |  |
| 1 | 83 | 33 | 39.8 | 28 | 33.7 | 22 | 26.5 | 107 | 17 | 15.9 | 47 | 43.9 | 43 | 40.2 |  |
|  |  |  |  |  |  |  |  |  |  |  |  |  |  |  |  |
| 2 | 65 | 19 | 29.2 | 32 | 49.2 | 14 | 21.5 | 61 | 18 | 29.5 | 22 | 36.1 | 21 | 34.4 |  |
| 3 | 82 | 48 | 58.6 | 23 | 28.1 | 11 | 13.4 | 62 | 19 | 30.7 | 14 | 22.6 | 29 | 46.8 |  |
|  |  |  |  |  |  |  |  |  |  |  |  |  |  |  |  |
| 4 | 116 | 65 | 56 | 37 | 31.9 | 14 | 12.1 | 114 | 29 | 25.4 | 26 | 22.8 | 59 | 51.8 |  |
|  |  |  |  |  |  |  |  |  |  |  |  |  |  |  |  |
| 5 | 107 | 62 | 57.9 | 29 | 27.1 | 16 | 15 | 73 | 12 | 16.4 | 22 | 30.1 | 39 | 53.4 |  |
|  |  |  |  |  |  |  |  |  |  |  |  |  |  |  |  |
| 6 | 65 | 36 | 55.4 | 24 | 36.9 | 5 | 7.7 | 68 | 18 | 26.5 | 14 | 20.6 | 36 | 52.9 |  |
|  |  |  |  |  |  |  |  |  |  |  |  |  |  |  |  |
| 7 | 47 | 29 | 61.7 | 12 | 25.5 | 6 | 12.8 | 40 | 11 | 27.5 | 10 | 25 | 19 | 47.5 |  |
|  |  |  |  |  |  |  |  |  |  |  |  |  |  |  |  |
| 8 | 28 | 15 | 53.6 | 11 | 39.3 | 2 | 7.1 | 38 | 22 | 57.9 | 4 | 10.5 | 12 | 31.6 |  |
|  |  |  |  |  |  |  |  |  |  |  |  |  |  |  |  |
| 9 | 145 | 57 | 39.3 | 49 | 33.8 | 39 | 26.9 | 127 | 16 | 12.6 | 38 | 29.9 | 73 | 57.5 |  |
|  |  |  |  |  |  |  |  |  |  |  |  |  |  |  |  |
| 10 | 56 | 13 | 23.2 | 28 | 50 | 15 | 26.8 | 105 | 18 | 17.1 | 34 | 32.4 | 53 | 50.5 |  |
|  |  |  |  |  |  |  |  |  |  |  |  |  |  |  |  |
| 11 | 42 | 26 | 61.9 | 12 | 28.6 | 4 | 9.5 | 78 | 18 | 23.1 | 24 | 30.8 | 36 | 46.2 |  |
|  |  |  |  |  |  |  |  |  |  |  |  |  |  |  |  |
| 12 | 251 | 133 | 53 | 76 | 30.3 | 42 | 16.7 | 94 | 15 | 16 | 25 | 26.6 | 54 | 57.5 |  |
|  |  |  |  |  |  |  |  |  |  |  |  |  |  |  |  |
| 13 | 99 | 45 | 45.5 | 33 | 33.3 | 21 | 21.2 | 88 | 18 | 20.5 | 21 | 23.9 | 49 | 55.7 |  |
|  |  |  |  |  |  |  |  |  |  |  |  |  |  |  |  |
| 14 | 34 | 26 | 76.5 | 8 | 23.3 | 0 | 0 | 85 | 14 | 16.5 | 26 | 30.6 | 45 | 52.9 |  |
|  |  |  |  |  |  |  |  |  |  |  |  |  |  |  |  |
|  |  |  |  |  |  |  |  |  |  |  |  |  |  |  |  |
| 15 | 34 | 16 | 47.1 | 10 | 29.4 | 8 | 23.5 | 54 | 18 | 33.3 | 14 | 25.9 | 22 | 40.7 |  |
|  |  |  |  |  |  |  |  |  |  |  |  |  |  |  |  |
| 16 | 91 | 45 | 49.5 | 32 | 35.2 | 14 | 15.4 | 72 | 35 | 48.1 | 15 | 20.8 | 22 | 30.6 |  |
|  |  |  |  |  |  |  |  |  |  |  |  |  |  |  |  |
| 17 | 77 | 27 | 35.1 | 26 | 33.8 | 24 | 31.2 | 87 | 31 | 35.6 | 28 | 32.2 | 28 | 32.2 |  |
|  |  |  |  |  |  |  |  |  |  |  |  |  |  |  |  |
| 18 | 133 | 60 | 45.1 | 57 | 42.9 | 16 | 12 | 118 | 65 | 55.1 | 24 | 20.3 | 29 | 24.6 |  |
|  |  |  |  |  |  |  |  |  |  |  |  |  |  |  |  |
| 19 | 63 | 13 | 20.6 | 41 | 65.1 | 9 | 14.3 | 98 | 24 | 24.5 | 2 | 2 | 72 | 73.5 |  |
|  |  |  |  |  |  |  |  |  |  |  |  |  |  |  |  |

**Table S3: Classification of items as red, amber and green items, by category in 2017 and 2021, as a percentage of all products**

|  | **2017** | | | | | | **2021** | | | | | |
| --- | --- | --- | --- | --- | --- | --- | --- | --- | --- | --- | --- | --- |
| **Category** | **Red** | | **Amber** | | **Green** | | **Red** | | **Amber** | | **Green** | |
|  | **n** | **% of all products** | **n** | **% of all products** | **n** | **% of all products** | **n** | **% of all products** | **n** | **% of all products** | **n** | **% of all products** |
| Bakery items (sweet and savoury) | 241 | 9.3 | 124 | 4.7 | 0 | 0 | 304 | 7.9 | 305 | 7.8 | 0 | 0 |
| Cold drinks | 326 | 12.3 | 122 | 4.6 | 55 | 2.2 | 196 | 5.0 | 574 | 14.6 | 99 | 2.6 |
| Fats and oils, spreads, sauces, dressings, condiments | 0 | 0 | 38 | 1.4 | 8 | 0.3 | 0 | 0 | 86 | 2.2 | 12 | 0.3 |
| Fish and other seafood, eggs, poultry, red meat (including processed meats) | 6 | 0.2 | 4 | 0.2 | 6 | 0.2 | 10 | 0.3 | 20 | 0.5 | 32 | 0.8 |
| Grain foods (bread, crackers, cereals) | 7 | 0.3 | 32 | 1.2 | 9 | 0.3 | 11 | 0.3 | 17 | 0.4 | 12 | 0.3 |
| Legumes, nuts, seeds | 38 | 1.4 | 67 | 2.5 | 33 | 1.2 | 38 | 1.0 | 209 | 5.3 | 54 | 1.4 |
| Milk and milk products (including milk alternatives) | 36 | 1.6 | 14 | 0.5 | 39 | 1.6 | 13 | 0.5 | 24 | 0.6 | 22 | 0.7 |
| Mixed dishes (packaged and unpackaged, sushi, sandwiches) | 235 | 8.9 | 348 | 13.1 | 145 | 5.5 | 108 | 2.7 | 546 | 13.9 | 446 | 11.4 |
| Packaged snack foods (including confectionery) | 598 | 22.5 | 26 | 1.0 | 0 | 0 | 457 | 11.6 | 168 | 4.3 | 0 | 0 |
| Vegetables and fruit | 14 | 0.5 | 0 | 0 | 74 | 2.8 | 10 | 0.3 | 8 | 0.2 | 135 | 3.4 |

NA: There are no criteria for green classification of bakery items or packaged snack foods in the Policy

**Table S4:** **Availability of items in the drinks category in 2017 and 2021**

|  | **2017** | | **2021** | |
| --- | --- | --- | --- | --- |
| **Category** | **n** | **% of drinks category** | **n** | **% of drinks category** |
| Coconut water | 11 | 2.2 | 1 | 0.1 |
| Energy drinks | 14 | 2.8 | 6 | 0.7 |
| Fruit and/or vegetable juices | 185 | 36.3 | 250 | 28.4 |
| Ice blocks | 19 | 3.7 | 3 | 0.3 |
| Still or carbonated flavoured drinks and milk drinks | 197 | 38.6 | 485 | 55.1 |
| Water | 77 | 15.1 | 124 | 14.1 |
| Milk-based smoothies | 7 | 1.4 | 12 | 1.4 |
| **Total beverages** | **510** | **100** | **881** | **100** |

**Table S5: Classification of cold drinks and packaged foods as red, amber and green items, by setting in 2017 and 2021**

|  | **2017** | | | | | | **2021** | | | | | |
| --- | --- | --- | --- | --- | --- | --- | --- | --- | --- | --- | --- | --- |
|  | **Red** | | **Amber** | | **Green** | | **Red** | | **Amber** | | **Green** | |
|  | **n** | **%** | **n** | **%** | **n** | **%** | **n** | **%** | **n** | **%** | **n** | **%** |
| **Cold Drinks *** | | | | | | | | | | | | |
| Food-service outlets | 212 | 63.9 | 89 | 26.8 | 31 | 9.3 | 182 | 37.3 | 246 | 50.4 | 60 | 12.3 |
| Vending machines | 119 | 66.9 | 33 | 18.5 | 26 | 14.6 | 20 | 5.1 | 328 | 84.1 | 42 | 10.8 |
| **Packaged snack foods** | | | | | | | | | | | | |
| Food-service outlets | 290 | 91.8 | 26 | 8.2 | NA | NA | 83 | 67.5 | 40 | 32.5 | 0 | 0 |
| Vending machines | 308 | 100 | 0 | 0 | NA | NA | 374 | 74.5 | 128 | 25.5 | 0 | 0 |

* Includes milk-based smoothies and ice-blocks
